# “Real-Life” Utility of the Graves’ Ophthalmopathy–Quality of Life in a Multidisciplinary Thyroid Eye Disease Service

**DOI:** 10.1097/IOP.0000000000002950

**Published:** 2025-04-16

**Authors:** Simrun Virdee, Malik Moledina, Vickie Lee

**Affiliations:** * Department of Ophthalmology, London North West University Healthcare NHS Trust; † Department of Ophthalmology, Imperial College & London North West University Healthcare NHS Trusts, London, United Kingdom

## Abstract

**Purpose:**

This study aimed to describe the correlation of demographic and clinical factors with the Graves’ ophthalmopathy–quality of life (GO-QOL) visual function (VF) and appearance (A) scores in a cohort attending a metropolitan multidisciplinary thyroid eye disease (TED) service.

**Methods:**

This is a cross-sectional retrospective study of 152 consecutive TED patients who completed the GO-QOL questionnaire. Clinical parameters, including endocrine diagnosis and status, TED activity and severity, Gorman diplopia score, and TED treatments were recorded at the time of completing each questionnaire and retrospectively analyzed.

**Results:**

A total of 257 GO-QOL questionnaires were completed over 32 months, with 59 patients providing sequential responses. The mean age was 50.0 ± 13.1 years, 77.0% (117/152) were female and 52.0% (79/152) were Caucasian. Graves’ disease was present in 86.2% (131/152) of participants, and 91.4% (139/152) were euthyroid at the time of the questionnaire. The mean time since TED onset was 2.5 years (range: 0.25–180 months). The mean ± standard deviation GO-QOL scores in the mild, moderate-to-severe, and sight-threatening disease cohorts for VF were 92.2 ± 15.2, 70.9 ± 28.6, and 56.6 ± 29.6, respectively, and for A were 79.1 ± 22.3, 48.2 ± 29.7, and 66.1 ± 35.3, respectively. VF and A scores were inversely correlated with clinical activity scores. Both scores improved postimmunosuppression but only A scores improved following decompression and rehabilitation surgery. Females and younger patients had lower A but not VF scores. East Asians and Caucasians had higher VF and A scores than African-Caribbeans.

**Conclusion:**

The GO-QOL’s granularity highlights the diverse functional and psychosocial experiences of TED patients, underscoring the need to integrate this valuable but underutilized tool into routine clinical practice.

## Abstract 2

This study provides a snapshot of the functional and psychosocial impact of thyroid eye disease and demonstrates significant demographic and clinical correlations of the Graves’ ophthalmopathy–quality of life in a “real-world,” ethnically diverse, UK-based metropolitan cohort.

Thyroid eye disease (TED) is a chronic, autoimmune inflammatory disease that can result in debilitating physical and psychological impairment with a consequential impact on quality of life (QOL).^1–3^ It is most commonly associated with Graves’ disease but can be associated with Hashimoto’s thyroiditis, euthyroidism, or hypothyroidism.^4,5^ About 25% to 50% of Graves’ disease patients suffer from TED; these patients may experience impaired vision (including irreversible sight loss) and/or altered appearance.^1,4,6^ Even though eye symptoms and signs can improve as inflammation subsides, both visual function and appearance can remain affected over the long term and can lead to impaired ability to work, high healthcare utilization, and significant direct and indirect costs.^7–9^ The significance of the psychological burden of TED should not be overlooked as these individuals have a significantly higher risk of death from suicide.^10^

The Graves’ ophthalmopathy–quality of life (GO-QOL) questionnaire was specifically developed^3^ to assess QOL in patients with TED, focusing on 2 key areas: (1) visual functioning, affected by double vision and reduced visual acuity, and (2) psychosocial effects resulting from altered appearance.^4^ These specific aspects were not covered by general health and vision-related QOL questionnaires.^6,11^ The GO-QOL has been proven to have content, construct, and cross-cultural validity, along with strong test–retest reliability and internal consistency.^1,2,4,6,11^ It is available in 13 languages and widely used in randomized control trials, including industry-sponsored trials of novel pharmaceutical agents.^1,4,12,13^ Despite the 2016 European Consensus recommendations for its routine clinical use,^14^ a UK survey found that only 21% of clinicians regularly collect patient-reported outcomes and QOL measures.^15^

The objective of this study was to investigate the correlation of demographic and clinical factors to GO-QOL scores in a “real-world,” ethnically diverse, UK-based metropolitan cohort. In addition, it aimed to assess the distribution of responses for various questions to provide a snapshot of the current state of dissatisfaction among TED patients.

## METHODS

This cross-sectional study included 152 consecutive patients attending multidisciplinary TED services (with co-location of endocrinology and ophthalmology consultants) based at the Imperial College Healthcare NHS Trust and London North West University Healthcare NHS Trust from February 2021 to October 2023. All patients underwent at least 1 endocrinology review to confirm the thyroid diagnosis (Graves’ disease, Hashimoto’s thyroiditis, hypothyroidism, or euthyroid), thyroid status (euthyroid, hyperthyroid, or hypothyroid), and treatment (medical management, radioiodine, thyroidectomy, or no treatment). TED activity (clinical activity score, CAS^16^), severity (EUGOGO criteria: mild, moderate-to-severe, or sight-threatening^17^), and diplopia (Gorman diplopia score^18^) were determined by a consultant ophthalmologist at each clinical visit. Details of previous, current, and planned treatments, including systemic immunosuppression, orbital radiotherapy, and decompression/rehabilitative surgery were also recorded and entered into the electronic patient records. The GO-QOL^3^ questionnaire was completed at each clinical encounter. GO-QOL scores and the above clinical parameters were retrospectively collected and analyzed. A total of 257 GO-QOL questionnaires were completed; where patients completed multiple questionnaires, this was also recorded. An elevated apparent diffusion coefficient of extraocular muscles on diffusion-weighted MRI was used as an imaging biomarker to predict activity.^19–21^

The English version of the GO-QOL questionnaire was incorporated into the electronic patient records where the scores were stored and later accessed to ascertain the responses to each question, which was scored on a 3-point Likert scale, as originally described by Terwee et al.^3^ The question evaluating the impact of disease on cycling was replaced by driving where applicable. The GO-QOL questionnaire of the appropriate language (downloaded from the EUGOGO website) was used for patients who understood limited English and was subsequently transposed into the electronic patient records. When an alternative questionnaire did not exist, a formal interpreter assisted in conducting the questionnaire (required in 2 questionnaires).

The study was approved by the Trust’s research ethics and audit committee with adherence to the tenets of the Declaration of Helsinki and all laws in the United Kingdom.

### Statistical Analysis

Continuous variables are expressed as means ± standard deviations and categorical variables are expressed as percentages. The ceiling and floor effects (the percentage of patients scoring the maximum and minimum score, respectively) of both GO-QOL subscales were considered significant if >15%, substantial if >30%, and excessive if >50% based on similar studies.^1,2,4,22^

The Shapiro-Wilk test, Kolmogorov-Smirnov test, and a histogram plot were used to assess the normality of the GO-QOL visual function (VF) and appearance (A) scores. GO-QOL subscale scores were not normally distributed so nonparametric tests were used in this study. The Mann-Whitney *U* test was used to test the significance between demographic/clinical covariates and the GO-QOL subscale scores. Where each covariate had more than 2 subcategories, a further subgroup analysis was performed using the Kruskal-Wallis rank-sum test. Spearman’s rank correlation coefficient was used to calculate the correlation between continuous variables (including patient age and proptosis) and the GO-QOL subscale scores.

With regards to immunosuppression as a covariate, the sample size for the “planned” (and therefore pre-) treatment group was too small to be analyzed independently so these patients were paired with those in the “continue” treatment group to represent the cohort of patients “currently undergoing” treatment. When comparing GO-QOL scores adjusted for disease severity among patients who had received different immunosuppressant medications, the mild and sight-threatening disease subgroups were excluded due to insufficient patient numbers.

Statistical analysis was conducted using a computerized software package (GraphPad) and a *p* value less than 0.05 was considered statistically significant.

## RESULTS

### Respondent Demographics and TED-related Characteristics

A total of 257 GO-QOL questionnaires were completed by 152 consecutive patients over 32 months, with 38.8% (59/152) providing sequential responses (Table 1). The cohort’s demographic and clinical data (Tables 2 and 3, respectively) showed that the mean age of patients was 50.0 ± 13.1 years, 77.0% (117/152) were female and 52.0% (79/152) were Caucasian. Among the participants, 86.2% (131/152) had Graves’ disease and 91.4% (139/152) were biochemically euthyroid at the time of completing the questionnaire. The mean interval from TED onset to the first recorded GO-QOL was 29.9 ± 30.4 months (range: 0.25–180 months). Overall, the mean GO-QOL scores were 78.7 ± 26.8 for VF and 61.3 ± 31.1 for A.

**TABLE 1.** Number of GO-QOL questionnaires completed by patients (total number of questionnaires = 257, total number of patients = 152)

No. questionnaires completed per patient	No. patients (N)	% of patients (%)
1	93	61.2
2	33	21.7
3	13	8.6
4	9	5.9
5	1	0.7
6	3	2.0

Single vs. multiple (≥2) questionnaire cohorts: no significant difference in disease activity (*p* = 0.582), more severe disease in multiple questionnaire cohort (*p* < 0.001).

GO-QOL, Graves’ ophthalmopathy-quality of life.

**TABLE 2.** Demographic data of patients included in the study (excluding repeated data from patients who completed multiple questionnaires, total number of patients = 152)

Age		
Mean ± SD, range (years)	50.0 ± 13.1, 21.0–82.3	
	No. patients (*n*)	% of patients (%)
Sex		
Female	117	77.0
Male	35	23.0
Ethnicity		
Caucasian	79	52.0
African-Caribbean	19	12.5
South Asian	16	10.5
East Asian	12	7.9
Other	26	17.1
Smoking status		
Current smoker	30	19.7
Ex-smoker	36	23.7
Never smoked	66	43.4
Unknown	20	13.2
Thyroid diagnosis		
Graves’ disease	131	86.2
Hashimoto’s thyroiditis	5	3.3
Hypothyroid	7	4.6
Euthyroid	7	4.6
Uncertain	2	1.3

**TABLE 3.** Clinical data of patients included in the study (including data from all questionnaires, N = 257)

	No. patients (N)	% of patients
Clinical activity score		
<3	231	89.9
0	113	44.0
1	74	28.8
2	44	17.1
≥3	26	10.1
3	19	7.4
4	5	1.9
5	0	0.0
6	2	0.8
≥7	0	0.0
Severity		
Mild	102	39.7
Moderate-to-severe	143	55.6
Sight-threatening	12	4.7
Gorman score		
0	145	56.4
1	47	18.3
2	26	10.1
3	39	15.2
Thyroid status		
Hyperthyroid	12	4.7
Euthyroid	238	92.6
Hypothyroid	5	1.9
Unknown	2	0.8
Thyroid treatment		
Titrate	111	43.2
Block and replace	29	11.3
Thyroxine	81	31.5
None	36	14.0
Thyroid plan		
Oral treatment (continue/change dose)	189	73.5
Thyroidectomy	43	16.7
Radioiodine	24	9.3
Other	1	0.4
Immunosuppression		
Planned	8	3.1
Continue	49	19.1
Previous	60	23.3
Never	140	54.5
CI	0	0.0
Orbital radiotherapy		
Planned	0	0.0
Continue	0	0.0
Previous	21	8.2
Never	236	91.8
CI	0	0.0
Urgent decompression surgery		
Planned	15	5.8
Previous	18	7.0
Never	224	87.2
CI	0	0.0
Rehabilitation surgery		
Planned	13	5.1
Previous	29	11.3
Never	214	83.3
CI	1	0.4
MRI findings		
Active	121	47.1
Inactive	65	25.3
Not done	71	27.6
Proptosis*		
<21 mm	69	42.3
≥21 mm	94	57.7
Mean ± SD, range (mm)	21.3 ± 3.0, 9.0–27.5	
Time since TED onset		
Mean ± SD, range (months)	29.9 ± 30.4, 0.25–180.0	

* Proptosis data are only available for 163 patients.

CI, contraindicated.

#### Patients Who Did Not Require Immunosuppression or Surgery.

A total of 120 GO-QOL questionnaires were completed by the cohort who did not require immunosuppression or surgery to treat their TED. Nearly all, (97.5%, 117/120) had inactive disease (CAS < 3) at the time of the questionnaire. Mild disease was observed in 66.7% (80/120), whereas 33.3% (40/120) had moderate-to-severe disease. Unsurprisingly, no patients in this cohort had sight-threatening disease. Diplopia (Gorman ≥ 1) was experienced by 29.2% (35/120). The mean GO-QOL scores were 87.2 ± 21.5 for VF and 64.4 ± 31.2 for A.

#### Patients Requiring Immunosuppression.

A total of 117 GO-QOL questionnaires were completed by the cohort who required immunosuppression as part of the management of their TED. At the time of completing the questionnaire, 19.7% (23/117) had active disease, with a mean CAS of 3.43 in this subgroup. Mild disease was noted in 13.7% (16/117), moderate-to-severe disease in 76.1% (89/117), and sight-threatening disease in 10.3% (12/117). Diplopia (Gorman ≥ 1) was experienced by 60.7% (71/117) and it was constant (Gorman 3) in 28.2% (33/117). Patients were either treated with intravenous methylprednisolone (IVMP) alone (19.7%, 23/117) or IVMP + mycophenolate mofetil (MMF) (80.3%, 94/117). The mean GO-QOL scores were 68.2 ± 29.4 for VF and 56.5 ± 31.1 for A. Patients who had completed immunosuppression had significantly higher VF and A scores than those currently undergoing immunosuppression (VF: 77.9 ± 23.2 vs. 58.0 ± 31.7, *p* < 0.001 and A: 62.5 ± 31.1 vs. 50.2 ± 29.9, *p* = 0.031). In addition, patients who had never received immunosuppression had significantly higher GO-QOL VF and A scores than those who had undergone or were currently undergoing immunosuppression (VF: 87.5 ± 20.7 vs. 68.8 ± 29.0, *p* < 0.001, A: 65.2 ± 30.5 vs. 56.4 ± 31.9, *p* = 0.035). The VF scores in patients that were treated with IVMP + MMF were significantly lower than in those treated with IVMP alone both overall and after adjusting for disease severity (moderate-to-severe disease: 62.1 ± 29.6 vs. 86.2 ± 31.3, *p* = 0.003). There were no significant differences in A scores between those treated with IVMP + MMF and those treated with IVMP alone (moderate-to-severe disease: 50.1 ± 28.7 versus 48.0 ± 31.3, *p* = 0.814).

#### Patients Requiring Urgent Decompression Surgery.

Eighteen GO-QOL questionnaires were completed by patients who had undergone urgent decompression surgery. As expected, all patients had sight-threatening disease preceding their surgery and 7 patients had dysthyroid optic neuropathy in the cohort. Fifteen questionnaires were completed predecompression surgery. Active disease (CAS ≥ 3) was present in 16.7% (3/18) at the time of the questionnaire, and diplopia (Gorman ≥ 1) was experienced by 66.7% (12/18). Immunosuppression therapy was either currently being taken or had been completed by 72.2% (13/18). The mean GO-QOL scores were 63.9 ± 23.0 for VF and 57.8 ± 37.0 for A. Postoperative VF scores were significantly lower compared with preoperative values and to patients not requiring surgery (59.0 ± 22.6 vs. 83.7 ± 12.7, *p* = 0.002 and 80.0 ± 27.2, *p* < 0.001, respectively). In those that had undergone urgent decompression surgery, there was no significant correlation between VF scores and the best-corrected visual acuity (*R* = 0.107, *p* = 0.769) or color vision measured by Ishihara plates (*R* = −0.361, *p* = 0.305). There was no significant difference in A scores from pre- to postdecompression surgery (42.9 ± 27.8 vs. 59.7 ± 36.0, *p* = 0.052).

#### Patients Requiring Elective Rehabilitation Surgery.

Thirty-three GO-QOL questionnaires were completed by patients who underwent elective rehabilitation surgery. At the time of completing the questionnaire, 12.1% (4/33) had a CAS ≥3. Mild disease was observed in 30.3% (10/33), 69.7% (23/33) had moderate-to-severe disease and no patients had sight-threatening disease. Diplopia (Gorman ≥ 1) was experienced by 51.5% (17/33). Within this cohort, 36.3% (12/33) had received immunosuppression and 21.2% (7/33) had undergone urgent decompression surgery. The mean GO-QOL scores were 72.0 ± 31.7 for VF and 60.2 ± 34.5 for A. Postrehabilitation surgery, patients had higher A scores than prerehabilitation surgery (61.0 ± 33.9 vs. 34.6 ± 26.7, *p* = 0.023). However, there was no significant difference in VF scores following rehabilitation surgery (74.6 ± 23.6 vs. 72.2 ± 32.9, *p* = 0.400).

### Correlation of Covariates With GO-QOL Scores

**FIG. 1.**
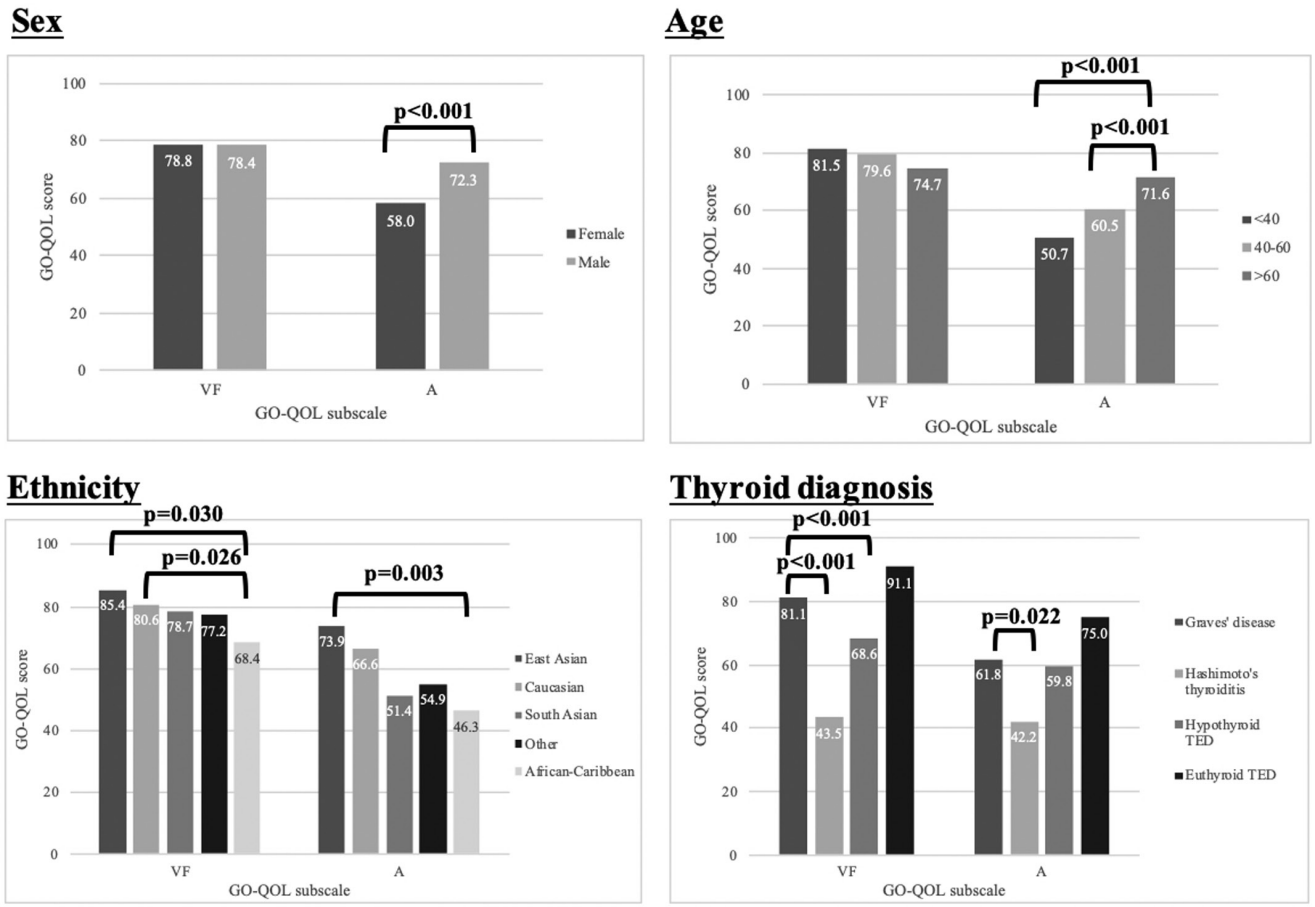
Correlation of demographic covariates with GO-QOL VF and A scores. A, appearance; GO-QOL, Graves’ ophthalmopathy-quality of life; VF, visual function.

**FIG. 2.**
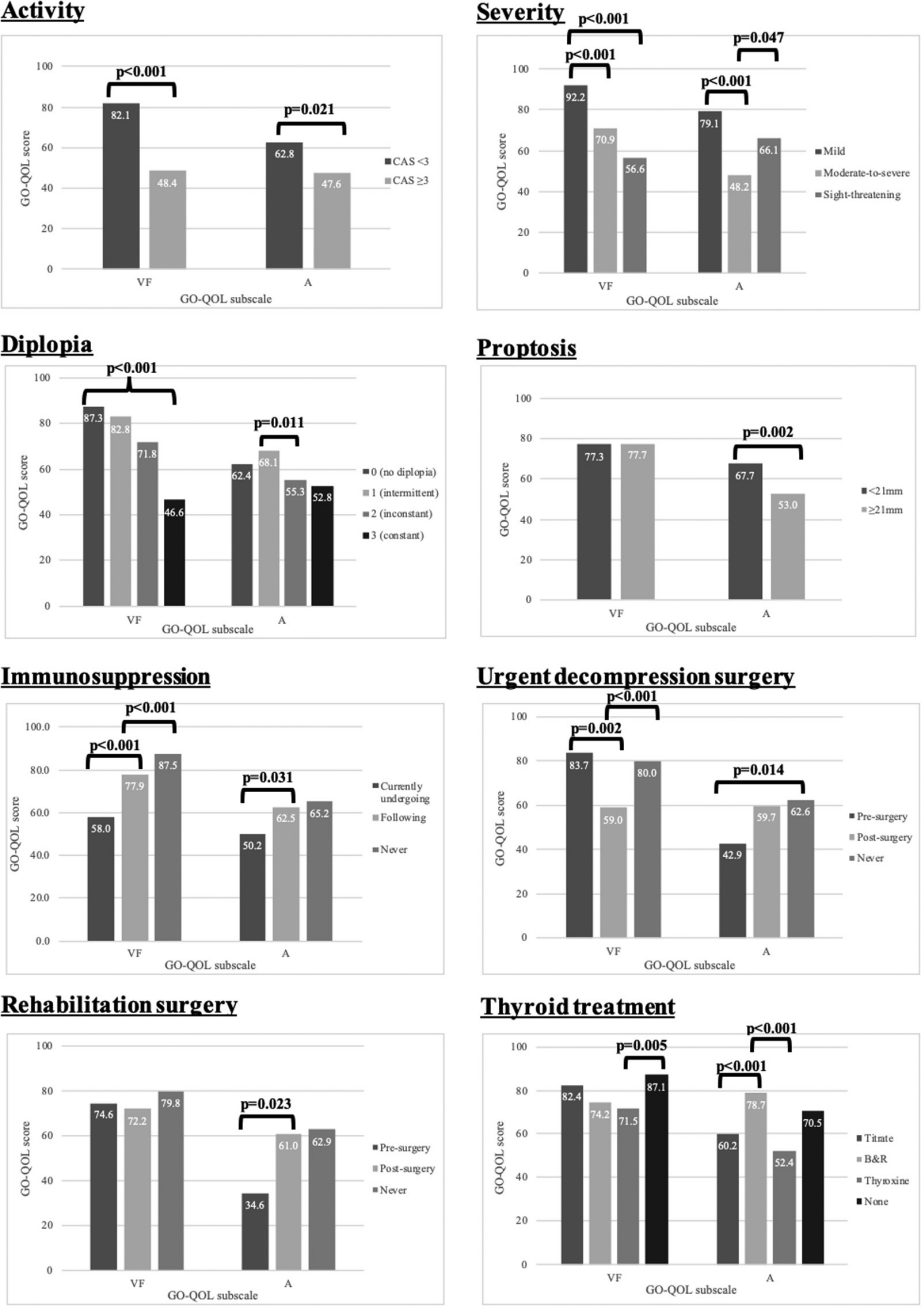
Correlation of clinical covariates with GO-QOL VF and A scores. A, appearance; GO-QOL, Graves’ ophthalmopathy-quality of life; VF, visual function.

The correlation of GO-QOL scores to demographic and clinical covariates is shown in Table 4 and displayed graphically in Figures 1 and 2, respectively.

**TABLE 4.** Correlation of demographic and clinical covariates with GO-QOL visual function and appearance scores

Covariate	Visual function (VF)	*p*	Appearance (A)		*p*
Age					
<40	81.5 ± 24.6	0.438	50.7 ± 31.9		**<0.001**
40–60	79.6 ± 26.0		60.5 ± 30.4		
>60	74.7 ± 29.5		71.6 ± 28.4		
	No statistically significant difference between subgroups		*p* < 0.001 between <40 vs. >60 and 40–60 vs. >60, *p* = 0.057 between <40 vs. 40–60		
	Spearman’s correlation coefficient: *R* = −0.117, *p* = 0.060		Spearman’s correlation coefficient: *R* = 0.264, *p* < 0.001		
Sex					
Female	78.8 ± 26.5	0.555	58.0 ± 30.7		**<0.001**
Male	78.4 ± 27.9		72.3 ± 29.8		
Ethnicity					
Caucasian	80.6 ± 24.0	0.179	66.6 ± 28.7		**0.003**
African-Caribbean	68.4 ± 30.1		46.3 ± 30.3		
South Asian	78.7 ± 28.1		51.4 ± 34.8		
East Asian	85.4 ± 22.5		73.9 ± 27.1		
Other	77.2 ± 31.3		54.9 ± 32.1		
	*p* = 0.030 between East Asian vs. African-Caribbean, *p* = 0.026 between Caucasian vs. African-Caribbean, no statistically significant difference between other subgroups		*p* = 0.007 between East Asian vs. African-Caribbean, *p* = 0.001 between Caucasian vs. African-Caribbean, *p* =0.044 between East Asian vs. Other, no statistically significant difference between other subgroups		
Smoking status					
Current smoker	83.9 ± 19.9	0.655	62.4 ± 30.4		0.973
Ex-smoker	79.3 ± 27.6		60.8 ± 31.5		
Never smoked	77.5 ± 27.4		62.0 ± 30.2		
Unknown	73.7 ± 30.7		57.8 ± 34.3		
	No statistically significant difference between subgroups		No statistically significant difference between subgroups		
Thyroid diagnosis					
Graves’ disease	81.1 ± 24.7	**<0.001**	61.8 ± 31.1		0.058
Hashimoto’s thyroiditis	43.5 ± 34.8		42.2 ± 29.9		
Hypothyroid	68.6 ± 22.2		59.8 ± 28.9		
Euthyroid	91.1 ± 14.2		75.0 ± 21.7		
Uncertain*	100.0 ± 0.0		93.8 ± 0.0		
	*p* < 0.001 between Graves’ disease vs. Hashimoto’s thyroiditis and Graves’ disease vs. Hypothyroid, *p* = 0.194 between Graves’ disease vs. euthyroid		*p* = 0.022 between Graves’ disease vs. Hashimoto’s thyroiditis, no statistically significant difference between other subgroups		
Clinical activity score (CAS)					
<3	82.1 ± 23.6	**<0.001**	62.8 ± 30.7		**0.021**
≥3	48.4 ± 33.3		47.6 ± 31.3		
	*p* < 0.001 between CAS 0–7, (no patients with CAS 5 or 7)		*p* < 0.001 between CAS 0–7, (no patients with CAS 5 or 7)		
Severity					
Mild	92.2 ± 15.2	**<0.001**	79.1 ± 22.3		**<0.001**
Moderate-to-severe	70.9 ± 28.6		48.2 ± 29.7		
Sight-threatening	56.6 ± 29.6		66.1 ± 35.3		
	*p* < 0.001 between mild vs. moderate-to-severe and mild vs. sight-threatening, *p* = 0.072 between moderate-to-severe vs. sight-threatening		*p* < 0.001 between mild vs. moderate-to-severe, *p* = 0.047 between moderate-to-severe vs. sight-threatening, *p* = 0.435 between mild vs. sight-threatening		
Diplopia (Gorman score)					
0	87.3 ± 21.3	**<0.001**	62.4 ± 32.1		0.082
1	82.8 ± 19.7		68.1 ± 28.1		
2	71.8 ± 22.2		55.3 ± 23.2		
3	46.6 ± 29.7		52.8 ± 32.8		
	*p* = 0.022 between 0 vs. 1, *p* = 0.023 between 1 vs. 2, *p* = 0.001 between 2 vs. 3, *p* < 0.001 between no diplopia vs. diplopia		*p* = 0.011 between 1 vs. 2, *p* = 0.294 between no diplopia vs. diplopia, no statistically significant difference between other subgroups		
Thyroid status					
Hyperthyroid	72.2 ± 27.1	0.423	51.0 ± 38.2		0.622
Euthyroid	78.6 ± 27.0		61.3 ± 30.8		
Hypothyroid	93.6 ± 6.2		70.0 ± 15.5		
Unknown	100.0 ± 0.0		93.8 ± 0.0		
	No statistically significant difference between subgroups		No statistically significant difference between subgroups		
Thyroid treatment					
Titrate	82.4 ± 25.4	**0.006**	60.2 ± 29.7		**<0.001**
Block & replace (B&R)	74.2 ± 27.7		78.7 ± 22.8		
Thyroxine	71.5 ± 29.0		52.4 ± 31.5		
None	87.1 ± 19.6		70.5 ± 31.9		
	*p* = 0.003 between titrate vs. thyroxine, *p* = 0.005 between thyroxine vs. none, *p* = 0.011 between titrate vs. B&R vs. thyroxine, *p* = 0.080 between oral treatment vs. none, no statistically significant difference between other subgroups		*p* < 0.001 between titrate vs. B&R vs. thyroxine vs. none and between titrate vs. B&R vs. thyroxine, *p* = 0.001 between titrate vs. B&R, *p* < 0.001 between B&R vs. thyroxine, *p* = 0.006 between thyroxine vs. none, *p* = 0.027 between titrate vs. none, *p* = 0.032 between oral treatment vs. none, no other statistically significant difference between subgroups		
Thyroid plan					
Oral treatment	78.6 ± 28.2	0.584	63.9 ± 30.7		**0.016**
Thyroidectomy	78.8 ± 22.3		48.7 ± 31.9		
Radioiodine	79.2 ± 23.2		65.6 ± 25.0		
Other†	83.3 ± 0.0		6.25 ± 0.0		
	No statistically significant difference between subgroups		*p* = 0.005 between oral treatment vs. thyroidectomy, *p* = 0.048 between thyroidectomy vs. radioiodine, *p* = 0.984 between oral treatment vs. radioiodine		
Immunosuppression					
Planned/continue	58.0 ± 31.7	**<0.001**	50.2 ± 29.9		**0.018**
Previous	77.9 ± 23.2		62.5 ± 31.1		
Never	87.5 ± 20.7		65.2 ± 30.5		
	*p* < 0.001 between planned/continue vs. previous, planned/continue vs. never, previous vs. never, previous vs. continue		*p* = 0.031 between planned/continue vs. previous, *p* = 0.002 between planned/continue vs. never, *p* = 0.029 between continue vs. previous, no statistically significant difference between other subgroups		
	Paired sample analysis (from sequential questionnaires): insufficient sample size		Paired sample analysis (from sequential questionnaires): insufficient sample size		
	Sample size for “planned” immunosuppression was too small (<10) so grouped with “continue”				
Orbital radiotherapy					
Previous	77.4 ± 22.1	0.289	50.3 ± 27.4		0.056
Never	78.8 ± 27.2		62.2 ± 31.2		
	No patients in the “planned” group				
Urgent decompression surgery					
Planned	83.7 ± 12.7	**<0.001**	42.9 ± 27.8		0.052
Previous	59.0 ± 22.6		59.7 ± 36.0		
Never	80.0 ± 27.2		62.6 ± 30.5		
	*p* = 0.002 between planned vs. previous, *p* < 0.001 between previous vs. never, *p* = 0.317 between planned vs. never		*p* = 0.014 between planned vs. never, no statistically significant difference between other subgroups		
Rehabilitation surgery					
Planned	74.6 ± 23.6	0.400	34.6 ± 26.7		**0.009**
Previous	72.2 ± 32.9		61.0 ± 33.9		
Never	79.8 ± 25.9		62.9 ± 30.3		
Contraindication‡	100.0 ± 0.0		75.0 ± 0.0		
	No statistically significant difference between subgroups		*p* = 0.023 between planned vs. previous, *p* = 0.002 between planned vs. never, *p* = 0.889 between previous vs. never		
MRI findings					
Active	75.1 ± 30.0	0.319	65.9 ± 30.4		0.066
Inactive	82.1 ± 21.8		57.3 ± 31.2		
Not done	81.8 ± 24.2		57.0 ± 31.0		
	No statistically significant difference between subgroups		*p* = 0.039 between active vs. not done, no statistically significant difference between other subgroups		
Proptosis					
<21mm	77.3 ± 25.6	0.509	67.7 ± 29.8		**0.002**
≥21mm	77.7 ± 28.8		53.0 ± 30.8		
	Spearman’s correlation coefficient: *R* = −0.015, *p* = 0.853		Spearman’s correlation coefficient: *R* = −0.367, *p* = 0		
Time since TED onset					
<15 months	83.3 ± 23.4	0.596	64.3 ± 29.1	0.313	
≥15 months	81.8 ± 22.0		59.3 ± 30.2		
	Spearman’s correlation coefficient: *R* = 0.017, *p* = 0.826		Spearman’s correlation coefficient: *R* = −0.034, *p* = 0.658		

Boldfaced value indicates *p* values less than 0.05 (statistically significant).

* Uncertain: not enough data to include in *p*-value testing and subgroup comparison.

† Other: not enough data to include in *p*-value testing and subgroup comparison.

‡ Contraindication: not enough data to include in *p*-value testing and subgroup comparison.

TED, thyroid eye disease.

#### Demographic Covariates

##### Ethnicity.

East Asians had the highest VF and A scores while African-Caribbeans scored lowest (VF: 85.4 ± 22.5 vs. 68.4 ± 30.1, *p* = 0.030, A: 73.9 ± 27.1. vs. 46.3 ± 30.3, *p* = 0.007).

##### Sex.

Females had lower A scores than males (58.0 ± 30.7 vs. 72.3 ± 29.8, *p* < 0.001) even after adjusting for disease activity, severity, and immunosuppression (Table 5). No correlation existed between sex and VF scores.

**TABLE 5.** Sex comparison of GO-QOL A scores, adjusted for disease activity, severity, and immunosuppression

	Female	*p*	Male
Clinical activity score (CAS)			
<3	59.6 ± 30.4	**<0.001**	73.9 ± 29.1
≥3	44.4 ± 30.1	0.395*	58.3 ± 32.6
Severity			
Mild	76.4 ± 23.7	**0.029**	88.6 ± 11.7
Moderate-to-severe	44.8 ± 27.6	**0.014**	59.8 ± 33.4
Sight-threatening	57.8 ± 38.4	-†	82.8 ± 19.5
Immunosuppression			
Planned/continue‡	44.4 ± 27.6	**0.014**	68.3 ± 29.6
Previous	59.1 ± 30.3	**0.018**	81.9 ± 28.3
Never	63.2 ± 30.5	0.097	71.4 ± 29.8

Boldfaced value indicates *p* values less than 0.05 (statistically significant).

* Sample size for male patients with CAS ≥3 is less than 10.

† Sample size for “sight-threatening” is too small to perform subgroup analysis.

‡ Sample size for “planned” immunosuppression too small (<10) so grouped with “continue”.

A, appearance; GO-QOL, Graves’ ophthalmopathy-quality of life.

##### Age.

Among patients with moderate-to-severe disease, younger patients had lower A scores than older patients (<40: 35.4 ± 25.6, 40–60: 46.3 ± 27.6, >60: 62.5 ± 30.4, *p* < 0.001, as shown in Table 6). No correlation existed between age and VF scores.

**TABLE 6.** Age comparison of GO-QOL VF and A scores, adjusted for disease severity

Visual function				
Severity	Age			*p*
	<40	40–60	>60	
Mild	90.5 ± 14.6	92.5 ± 16.7	93.3 ± 10.5	0.783
Moderate-to-severe	75.9 ± 27.7	70.5 ± 26.6	67.3 ± 32.0	0.332
Sight-threatening	-*	43.8 ± 28.5	63.1 ± 28.0	-†
**Appearance**				
**Severity**	**Age**			***p***
	**<40**	**40–60**	**>60**	
Mild	75.0 ± 25.2	78.9 ± 21.6	83.6 ± 19.9	0.399
Moderate-to-severe	35.4 ± 25.6	46.3 ± 27.6	62.5 ± 30.4	**<0.001**
Sight-threatening	-*	28.1 ± 31.1	85.2 ± 17.4	-†

A statistically significant difference is observed between mild vs. moderate-to-severe vs. sight-threatening within each age category. Boldfaced value indicates *p* values less than 0.05 (statistically significant).

* No patients with sight-threatening disease <40 years old.

† Sample size for “sight-threatening” is too small to perform subgroup analysis.

A, appearance; GO-QOL, Graves’ ophthalmopathy-quality of life; VF, visual function.

#### Clinical Covariates

##### Activity.

Patients with active disease had lower VF and A scores than those with inactive disease (VF: 48.4 ± 33.3 [CAS ≥ 3] vs. 82.1 ± 23.6 [CAS < 3], *p* < 0.001, A: 47.6 ± 31.3 [CAS ≥ 3] vs. 62.8 ± 30.7 [CAS < 3], *p* = 0.021).

##### Severity.

The GO-QOL VF scores in the mild versus moderate-to-severe/sight-threatening (nonmild) cohorts were 92.2 ± 15.2 versus 69.8 ± 29.0, *p* < 0.001. The GO-QOL A scores in the mild versus moderate-to-severe/sight-threatening (nonmild) cohorts were 79.1 ± 22.3 versus 49.6 ± 30.5, *p* < 0.001. Unlike VF scores which decreased with increasing disease severity, patients with moderate-to-severe disease had lower A scores than those with mild disease and sight-threatening disease (48.2 ± 29.7 vs. 79.1 ± 22.3, *p* < 0.001 and 66.1 ± 35.3, *p* = 0.047 respectively).

##### Diplopia.

A negative correlation existed between the degree of diplopia and VF scores, whereas for appearance, a significant difference was only found between Gorman scores 1 and 2 (68.1 ± 28.1 vs. 55.3 ± 23.2, *p* = 0.011). The VF and A scores were significantly higher in the Gorman 0/1 cohort compared with the Gorman 2/3 cohort (VF: 86.2 ± 21.0 vs. 56.7 ± 29.7, *p* < 0.001, A: 63.8 ± 31.3 vs. 53.8 ± 29.4, *p* = 0.0139). Among drivers, 18.1% (21/116) had gaze-evoked or constant diplopia (Gorman score ≥ 2).

##### Proptosis.

There was a negative correlation between A scores and the degree of proptosis (67.7 ± 29.8 [<21 mm] vs. 53.0 ± 30.8 [≥21 mm], *p* = 0.002, *R* = −0.367, *p* = 0). There was no correlation between VF scores and proptosis (77.3 ± 25.6 [<21 mm] vs. 77.7 ± 28.8 [≥21 mm], *p* = 0.509, *R* = −0.015, *p* = 0.853).

##### Disease duration.

No correlation was observed between GO-QOL VF and A scores and disease duration. VF: 83.3 ± 23.4 (<15 months) vs. 81.8 ± 22.0 (≥15 months), *p* = 0.596, A: 64.3 ± 29.1 (<15 months) vs. 59.3 ± 30.2 (≥15 months), *p* = 0.313.

##### MRI activity.

No correlation was observed between GO-QOL VF and A scores and MRI activity. VF: 75.1 ± 30.0 (MRI active) vs. 82.1 ± 21.8 (MRI inactive), *p* = 0.276, A: 65.9 ± 30.4 (MRI active) vs. 57.3 ± 31.2 (MRI inactive), *p* = 0.080.

##### Thyroid status.

There was no correlation between thyroid status and VF scores (*p* = 0.423) or A scores (*p* = 0.622).

The proportion of patients with a GO-QOL score of ≤75, indicating a substantial impairment of QOL,^23^ adjusted for disease severity was as follows: mild disease—VF 11.8% (12/102) and A 34.3% (35/102); moderate-to-severe disease—VF 45.5% (65/143) and A 80.4% (115/143); sight-threatening disease—VF 75.0% (9/12) and A 41.7% (5/12).

There was a substantial ceiling effect for the VF subscale as 40.1% (103/257) achieved the maximum score. Among those with mild disease only, 64.7% (66/102) achieved the maximum score. There was no significant floor effect on the VF or A subscale.

### GO-QOL Questionnaire Responses

**FIG. 3.**
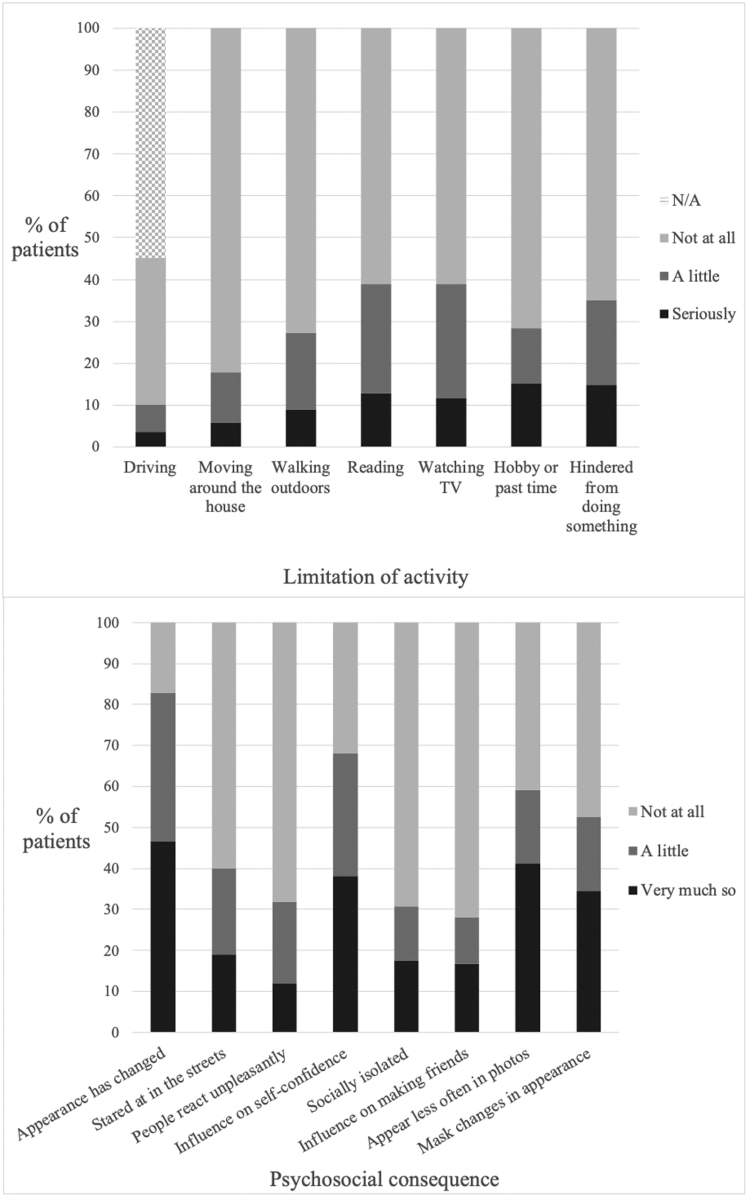
Stacked bar chart showing the frequencies of responses to each item of the GO-QOL VF (above) and A (below) questionnaires. A, appearance; GO-QOL, Graves’ ophthalmopathy-quality of life; VF, visual function.

**FIG. 4.**
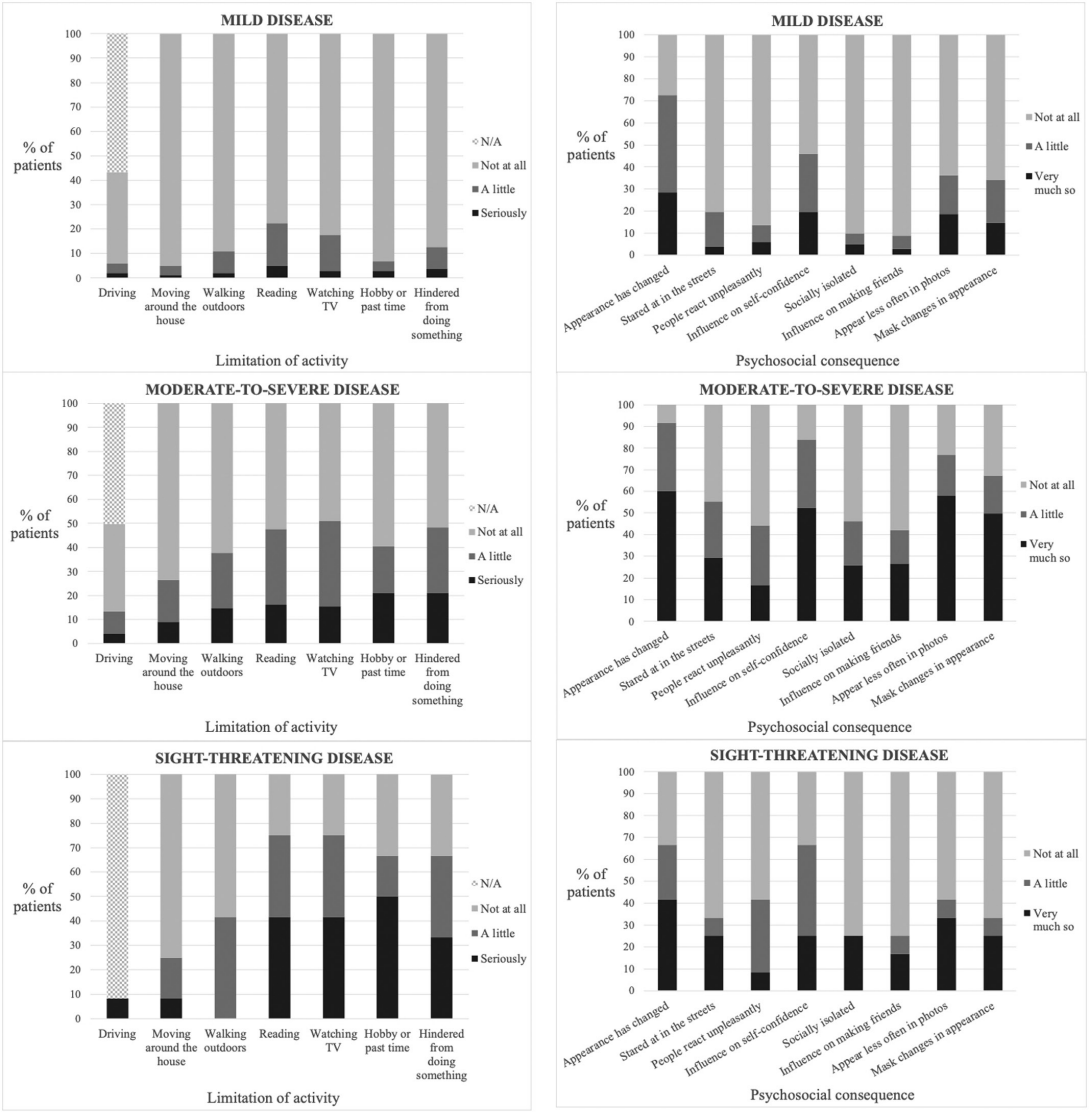
Stacked bar chart showing the frequencies of responses to each item of the GO-QOL VF (left) and A (right) questionnaires adjusted for disease severity (mild [above], moderate-to-severe [middle], sight-threatening [below]). A, appearance; GO-QOL, Graves’ ophthalmopathy-quality of life; VF, visual function.

The most frequently limited activities were reading (38.9%, 100/257), watching TV (38.9%, 100/257), and feeling hindered from doing something (35.0%, 90/257), as shown in Table 7 and Figure 3. Among patients with sight-threatening disease, 91.7% (11/12) reported that they “do not drive” and of these, 63.6% (7/11) were undergoing active immunosuppression. The most frequent psychosocial sequelae were experiencing a change in appearance (82.9%, 213/257) and an influence on self-confidence (68.1%, 175/257). In clinically mild disease, 72.5% (74/102) still felt that their appearance had changed (Fig. 4).

**TABLE 7.** Frequencies of responses to each item of the GO-QOL questionnaire, % (n = 257)

Visual function subscale (functional impact)	Seriously	A little	Not at all	N/A
Driving	3.5 (7.8)	6.6 (14.6)	35.0 (77.6)	54.9*
Moving around the house	5.8	12.1	82.1	-
Walking outdoors	8.9	18.3	72.8	-
Reading	12.8	26.1	61.1	-
Watching TV	11.7	27.2	61.1	-
Hobby or pastime	15.2	13.2	71.6	-
Hindered from doing something	14.8	20.2	65.0	-
**Appearance subscale (psychosocial impact**)	**Very much so**	**A little**		**Not at all**
Appearance has changed	46.7	36.2		17.1
Stared at in the streets	19.1	21.0		59.9
People react unpleasantly	12.1	19.8		68.1
Influence on self-confidence	38.1	30.0		31.9
Socially isolated	17.5	13.2		69.3
Influence on making friends	16.7	11.3		72.0
Appear less often in photos	41.2	17.9		40.9
Mask changes in appearance	34.6	17.9		47.5

The values inside the parenthesis indicate frequencies of responses excluding patients who do not drive, % (N = 116). Bicycling is not included in the UK version of the questionnaire.

* Not applicable as these patients do not drive.

GO-QOL, Graves’ ophthalmopathy-quality of life.

## DISCUSSION

It is generally acknowledged that there is a gap between the TED patients’ lived experience and the clinical classification of activity and severity, which can be partially bridged with disease-specific QOL questionnaires. In most contexts, the GO-QOL has been reported as an outcome measure following specific TED interventions. However, its use in daily clinical practice remains infrequent, despite recommendations by the 2016 European Consensus^14^ recommending its routine use. This underutilization may partly reflect perceptions of the questionnaire’s extensive nature. Nevertheless, a UK survey revealed that only 21% of respondents routinely collected patient-reported outcomes and QOL measurements,^15^ suggesting that this trend is more indicative of general clinical practices rather than a specific limitation of the GO-QOL, such as its length or complexity. This is the first study, to our knowledge, to explore the correlation of demographic and clinical factors to background GO-QOL scores in a “real-life” UK-based TED cohort.

Our cohort mostly consisted of patients with clinically inactive, moderate-to-severe disease, a profile similar to that found in other population studies.^1–4,6,11^ The mean VF score was higher in this study (78.7) compared with others (Korean: 73.7, German: 72.5, Chinese: 68.4, Australian: 59.0, Taiwanese: 58.4, and Dutch: 54.7).^1–4,6,11^ The mean A score was comparable with other populations (61.3 in this study, German: 71.3, Chinese: 62.0, Korean: 61.9, Dutch: 60.1, and Taiwanese and Australian: 54.5).^1–4,6,11^ This indicates greater variability in functional impairment than psychosocial impairment across populations, likely influenced by geographical differences in treatment access^24^ and ethnic variations in disease manifestation.

Our study supports a correlation between ethnicity and GO-QOL scores, with East Asians and Caucasians scoring higher on both functional and psychosocial aspects than South Asians and African-Caribbeans. This observation aligns with previous studies and may be attributed to generally lesser proptosis^25^ in East Asians due to ethnic variations in orbital anatomy. African-Caribbeans have shallower orbits, increased orbital soft tissue, and reduced malar prominence and TED can exaggerate the appearance of proptosis.^26^ Furthermore, the African-Caribbean cohort generally presents with fewer clinical features on current classification guidelines (which have been validated on Caucasian populations) yet still require longer follow-ups, similar treatments and do not have better outcomes.^27^ It should be noted that the majority of international studies involve East Asian and Caucasian populations while there is relatively limited research on TED in South Asians and African-Caribbeans.

We observed a negative correlation between both GO-QOL subscale scores and disease activity, consistent with previous reports.^1,2,6,11^ Similarly, we identified a negative correlation between VF scores and disease severity. Patients with moderate-to-severe disease scored lower on the A subscale than those with mild or sight-threatening disease. Although the correlation between GO-QOL subscale scores and disease severity, as well as the discrepancy between VF and A scores following decompression surgery, have been previously reported,^28,29^ our observation that individuals with moderate-to-severe disease score lower on the A subscale compared with those with mild or sight-threatening disease has not been previously documented.^1,2,4,6^ This may be partially explained by the high prevalence of urgent orbital decompression (reducing proptosis) in the sight-threatening disease cohort. Another possible explanation is that patients with sight-threatening disease do not necessarily have significant proptosis or eyelid retraction and, therefore, may have better A scores compared with those with moderate-to-severe disease. In cases of clinically mild disease, very few patients scored below 50, indicating a minimal floor effect. However, there was an excessive ceiling effect for VF and a significant ceiling effect for A, with 64.7% and 18.6%, respectively, achieving the maximum score. This suggests that the GO-QOL may lack sensitivity in detecting improvements in patients with milder forms of the disease.

Patients requiring immunosuppression were treated according to EUGOGO guidelines with IVMP alone, IVMP + MMF, or IVMP + orbital radiotherapy. Prior to the 2021 guidelines,^14^ IVMP was the primary treatment with second-line therapies such as MMF or orbital radiotherapy reserved for more severe disease manifestations, such as significant diplopia. This likely explains the significantly lower VF scores observed in the IVMP + MMF group compared with the IVMP alone group in our study. Following the 2021 guidelines,^17^ most patients received concurrent IVMP + MMF with the duration of MMF tailored to disease severity. While variations in immunosuppressive regimens complicate QOL outcome comparisons across patients, these reflect the dynamic, “real-world” implementation of evolving treatment protocols.

As expected, diplopia (measured by the Gorman score) negatively correlated with VF scores but not with A scores which aligns with most previous studies.^1,4,6,11^ Patients who have undergone urgent decompression surgery had significantly lower VF scores compared with presurgery scores which may reflect the ongoing visual morbidity of diplopia and visual dysfunction. Although our cohort of patients with dysthyroid optic neuropathy was small (only 7 patients), the absence of significant correlation between VF scores and both best-corrected visual acuity and Ishihara color vision scores suggests that factors beyond visual acuity recovery, such as severe diplopia, may contribute to poor visual function following urgent decompression surgery. Scores improved following decompression surgery, although the change did not reach statistical significance, likely due to the sample size. This finding is consistent with another study that observed improvements in A scores earlier than VF scores.^13^ In our cohort, the mean time since TED onset was 2.5 years, yet patients continued to have low QOL even after 15 years. This correlates with a previous study of 120 TED patients with a median follow-up of 10 years; 32% of patients reported that their eyes still did not appear normal and 28% were not satisfied with the appearance of their eyes^30^ demonstrating the chronic psychological toll on TED patients.

We found that elective rehabilitation surgery improved A scores. In contrast, VF scores were high and showed no significant change postsurgery, suggesting a “response shift” in patients’ internal standards, values, and self-perception of QOL,^31^ particularly in those with inactive and stable disease. We observed a negative correlation between the extent of proptosis and A scores but not VF scores, consistent with other studies.^2,4,6^

MRI activity did not correlate with either GO-QOL subscale, suggesting that radiological imaging may not be a reliable predictor of QOL, a finding not previously reported. Notably, we observed a discrepancy between the proportion of patients classified as having active disease based on CAS (10.1%) versus MRI findings (47.1%). An elevated apparent diffusion coefficient of the extraocular muscles on diffusion-weighted MRI was used as an imaging biomarker to predict activity, which has been shown to have a positive association with CAS (*p* ≤ 0.001)^20^ and reported to have an 87% sensitivity in identifying active TED.^19^ The above discrepancy could be attributed to the possibility that elevated apparent diffusion coefficient values serve as an early biomarker of asymptomatic extraocular muscle inflammation in TED,^32^ appearing before or even in the absence of clinical signs. In addition, it may reflect our multiethnic cohort, as non-Caucasian patients often present with fewer clinical features under current classification guidelines, a limitation of conventional clinical assessment tools for TED.^20^

Similarly, smoking status showed no correlation with QOL scores in our study, differing from the findings in the German study that reported lower A scores among smokers.^2^

Graves’ disease patients had higher scores on both subscales than those with Hashimoto’s thyroiditis (lowest scores) and hypothyroid TED (for VF only), which may be explained by a delayed diagnosis in non-Graves’ disease subtypes. However, caution is warranted when interpreting these results due to the influence of multiple confounding variables.

Females had lower A scores than males, even after adjusting for CAS, disease severity, and immunosuppression status, indicating a gender impact on appearance and psychosocial well-being. This trend is consistent with most international studies.^1,2,4,33^ We found no correlation between gender or age and VF scores, even after adjusting for disease severity, whereas a positive correlation was found between age and A scores for patients with moderate-to-severe disease, again mostly consistent with previous reports.^3,4,11^

### Limitations of This Study and Suggestions for Further Research

As this is a cross-sectional study, we did not evaluate changes over time and cannot establish causal relationships. Longitudinal studies would help identify causal factors. While 38.8% of participants completed more than 1 questionnaire, we lacked sufficient data for paired statistical analysis. In addition, individuals who completed multiple questionnaires tended to have more severe disease, despite exhibiting similar disease activity, which could indicate a potential confounding factor. We had limited data for certain patient groups: young patients (under the age of 40), those with sight-threatening disease, and patients planning to commence therapies (which limits our conclusion on treatment effects). We also did not specifically address and record asymmetrical TED. Further work focusing on these groups would be beneficial. Our data were not adjusted for previous treatments nor the timing of treatments; this could yield a heterogenous response in subgroups, which may not reflect the true QOL and may make comparisons of changes in QOL after treatment difficult to interpret. Furthermore, there was no uniform schedule for checking the GO-QOL; rather, questionnaires were performed at each clinical encounter so an associated bias cannot be excluded. While the involvement of multiple ophthalmologists helped mitigate systemic bias, the possibility of inter-observer variability cannot be excluded. In addition, neither the assessing ophthalmologist nor the participants were blinded so associated biases may be present.

It should also be noted that the GO-QOL questionnaire may overlook certain psychosocial impacts due to its standardized structure. A qualitative approach can provide deeper insights into these experiences. For instance, Wiersinga identified 3 recurring themes through a qualitative analysis of patients’ lived experiences: (1) altered identity, (2) coping strategies, and (3) challenging interactions with healthcare providers.^9^

## CONCLUSION

Our study is the first to explore the correlation of GO-QOL scores with demographic and clinical factors in a multicentric UK-based TED cohort. Our comprehensive analysis of covariates introduces new insights into factors affecting QOL in TED patients. Conducted in diverse inner-city healthcare institutions across London, it provides a unique multiethnic perspective, which is particularly valuable for understudied South Asian and African-Caribbean populations. The GO-QOL’s granularity highlights the diverse functional and psychosocial experiences of these patients, underscoring the need to integrate this valuable but underutilized tool into routine clinical practice.
